# The Royal British Nurses' Association

**Published:** 1897-11-27

**Authors:** 


The Hospital

A Journal of

The Medical Sciences and Hospital Administration.

Vol. XXIII.—No. 583.] Edited by Sir Henry Burdett, K.C.B., and Solomon C. Smith, M.D., M.R.C.P. [Nov. 27, 1897.

### The Royal British Nurses' Association.

The Royal British Nurses' Association, which for the last few years has been struggling against difficulties mainly occasioned by defects in its constitution, and by the facilities which these defects have afforded to the pursuit of an obstructive policy by a small minority of its members, has at last taken a decided and practical step in the direction of a substantial reform. The majority of the founders of the Nurses' Association thought it desirable to make provision for the periodical infusion of new blood into its councils and committees; and this was done, or at least was attempted, by a rule that one-third of the members of these bodies should retire annually, and that the members so retiring should not be eligible for re-election until after the lapse of one year from the time of their retirement. The provision is an ordinary one, and its wisdom has been confirmed by much experience. In the Nurses' Association, however, there were some who desired to see this rule made subject to exceptions, so that there should be members of the ruling bodies whose positions were permanent, and others whose positions were temporary. Such an arrangement as this has also been tried in other societies, and its invariable result has been that the permanent members have obtained complete control over affairs, and that the functions of the temporary members have been little more than nominal. When the time came for the retirement of a third part of the members of the Council and of the Committee of the Association, it was found that serious differences of opinion existed as to the proper interpretation of the charter and bye-laws in this respect, and that such differences were not wholly absent from the minds of some of the learned counsel who were appealed to on the subject. But the preponderance of legal opinion was decidedly in favour of the general applicability of the retirement rule, which was accordingly enforced, although its enforcement has ever since been put forward as a substantial grievance by some of those who were affected by it. The alleged ambiguity about retirement was by no means the only one which could be discovered, and the Association has thus been brought face to face with the necessity for a complete reconstruction of its bye-laws, and has taken the opportunity not only of rendering them clear, but also of so modifying them as to supplycertain needs which time and circumstances have disclosed. The proposed new laws, after having been carefully considered and discussed in the Executive Committee, and after having been approved by counsel, were submitted last week to a very full meeting of the Council of the Association, at which Her Royal Highness the President was present, and which was held in the large room of the Medical Society of London. Their adoption was opposed by a member who stood alone in his opposition, and who excited a good deal of irreverent merriment by his declaration that the retirement of certain members of the governing body of the Association had struck a serious blow at the integrity of the British Empire. Statesmen and philanthropists, he averred, had for years been striving to draw more closely the bonds between Great Britain and her colonies, and it was fondly expected that the occurrences of the Jubilee year would render material assistance to their efforts. But, at the critical moment, when all men's hopes ran high, the Executive Committee, or the Council, of the Royal British Nurses' Association intervened, and a return to chaos was the consequence. The nurses of Canada and the nurses of New Zealand refused to become affiliated to an association which was not governed by a partially permanent committee, and the Empire ceased to be a coherent and an united body. The speaker rendered it plain, however, that the mischief was not only done, but done beyond recall; and, possibly for this reason, the rest of the meeting thought it useless to cry over spilt milk, and wise, on the whole, to accept the proposed new bye-laws for better or worse. They will speedily be brought before a special general meeting of the Association convened for the purpose; and, if again accepted, will be sent for confirmation to the Privy Council. Their chief effect will be to reconstitute both the Council and the Executive Committee in such a way as to make the former less unwieldy, and both more truly representative than at present. The Council will be diminished in number, and will contain a much increased proportion of the matrons of important hospitals, who will sit

*ex-officio* instead of by election. The elected members, whether doctors, matrons, or nurses, will be subject to the rule of retirement. The whole of the Executive Committee, except the President, will retire and be elected annually, but the retiring members will be eligible for re-election. We trust that the new laws will soon be brought into operation, and that, under their influence, the Association may enter upon a career of wider usefulness than has hitherto been within its reach.

